# High pressures increase α-chymotrypsin enzyme activity under perchlorate stress

**DOI:** 10.1038/s42003-020-01279-4

**Published:** 2020-10-02

**Authors:** Stewart Gault, Michel W. Jaworek, Roland Winter, Charles S. Cockell

**Affiliations:** 1 UK Centre for Astrobiology, SUPA School of Physics and Astronomy, University of Edinburgh, James Clerk Maxwell Building, Peter Guthrie Tait Road, Edinburgh, EH9 3FD UK; 2 Faculty of Chemistry and Chemical Biology, Physical Chemistry I – Biophysical Chemistry, TU Dortmund University, Otto-Hahn-Str. 4a, D-44227 Dortmund, Germany

**Keywords:** Enzymes, Proteins

## Abstract

Deep subsurface environments can harbour high concentrations of dissolved ions, yet we know little about how this shapes the conditions for life. We know even less about how the combined effects of high pressure influence the way in which ions constrain the possibilities for life. One such ion is perchlorate, which is found in extreme environments on Earth and pervasively on Mars. We investigated the interactions of high pressure and high perchlorate concentrations on enzymatic activity. We demonstrate that high pressures increase α-chymotrypsin enzyme activity even in the presence of high perchlorate concentrations. Perchlorate salts were shown to shift the folded α-chymotrypsin phase space to lower temperatures and pressures. The results presented here may suggest that high pressures increase the habitability of environments under perchlorate stress. Therefore, deep subsurface environments that combine these stressors, potentially including the subsurface of Mars, may be more habitable than previously thought.

## Abstract (web summary)

Gault et al. show that high pressures increase α-chymotrypsin enzyme activity in the presence of high perchlorate concentrations. These perchlorate salts shift the folded enzyme phase space to lower temperatures and pressure and may move the optimum enzyme activity towards lower temperatures in addition to higher pressures, which has implications for Martian habitability.

## Introduction

When searching for habitable environments elsewhere in the cosmos, one criterion is to seek liquid water. This is well grounded in our understanding of the importance of an appropriate solvent for the processes of life to occur in ref. ^1^. Therefore on Mars, much attention has been paid to environments where evidence of liquid water has been observed or is predicted to exist^2,3^. However, the mere presence of liquid water does not make an environment habitable. How life responds to external physical and chemical factors such as temperature, pressure, pH, and salinity will also define the edges of habitability^4,5^.

On Mars, prime candidates for habitable environments are deep aqueous environments. These may take the form of the reported subglacial lake at the Martian south pole^3^, or the deep groundwater beneath the Martian cryosphere^2^. These cold environments have been hypothesised to contain high concentrations of perchlorate salts, following their detection at the surface^6^, due to their extremely low eutectic temperatures. Perchlorate salts have been shown to have deleterious effects on microbial life^7,8^ and so must be considered as a factor that will shape the habitability of these environments. In addition to perchlorate salts, sulphates are widely distributed across the Martian surface^9^ and so provide a useful comparison for Mars relevant salt effects on biochemistry. The Martian groundwater would experience pressures of ∼100 MPa (1 kbar) at the base of the cryosphere at the Martian poles if it reached a depth of 10 km^2^. Thus, to be able to assess the habitability of subsurface environments, we must investigate the combination of strong ionic effects and high pressures. Though there is quite a lot known about the effects cellular organic osmolytes, such as trimethylamine-*N*-oxide, impose on organisms thriving in the deep sea under high-pressure stress up to the 1000 bar level^10–12^, the combined effects of high salt, low temperature, and high pressure on biochemical processes is still terra incognita.

Previous studies have demonstrated the deleterious effects of destabilising ions such as perchlorates on the activity of α-chymotrypsin (α-CT) and other enzymes^7,13^. Conversely, stabilising salts, such as sodium sulphate^14^, have been shown to increase the activity and structural stability of α-CT^15^. Our knowledge of how these molecules affect enzyme activity is largely restricted to ambient temperatures and pressures. Therefore, a greater understanding of how these effects change with pressure is yet to be achieved.

The effect of pressure on enzyme kinetics is largely dictated by Le Châtelier’s principle. If an enzymatic reaction exhibits a negative change in volume (Δ*V* < 0), or negative activation volume (Δ*V*^‡^ < 0), the overall reaction yield and rate will increase in that direction. A negative volume change is achieved when the volume occupied by the enzyme-substrate complex (ES) is lower than that of the enzyme (E) and substrate (S) in solution. The same holds true for negative activation volumes, which occur when the activated transition state (TS) occupies a lower volume than the ES complex. Previous studies have shown that α-CT exhibits a negative activation volume and, as such, its activity increases with pressure^16–18^.

In this work, the effects that Mars relevant salts (MgSO_4_ and Mg(ClO_4_)_2_) and high hydrostatic pressures exert on the activity and structural stability of α-CT, an archetype of a digestive enzyme, are explored. Assayed concentrations of MgSO_4_ and Mg(ClO_4_)_2_ of 0.25 and 0.5 M allow for a comparison of the effects of both salts on our system as concentrations of Mg(ClO_4_)_2_ > 1 M induce unfolding of α-CT at room temperature. This work advances our understanding of how the interactions of high concentrations of these ions and high pressure influence the biochemistry and habitability of terrestrial and extraterrestrial environments.

## Results

### α-chymotrypsin activity in Mars salts

**Fig. 1:**
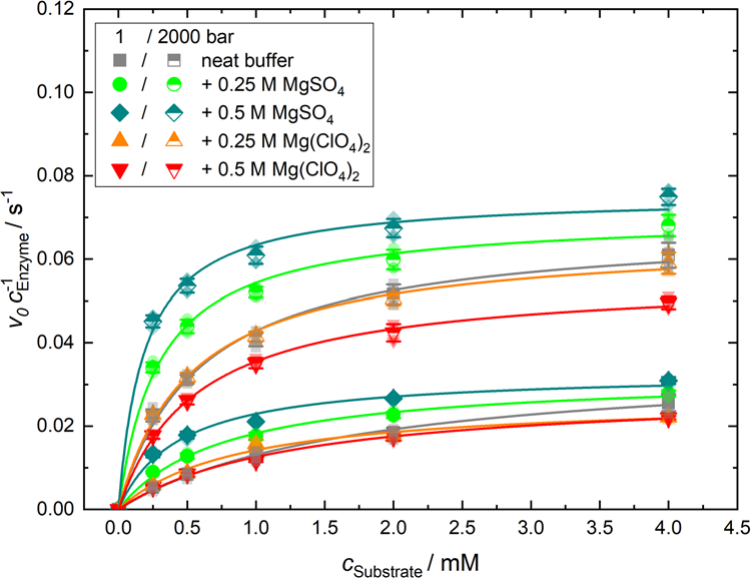
α-Chymotrypsin activity curves. Michaelis–Menten plots of the enzymatic activity of α-CT at *T* = 20 °C in different buffer solutions at two selected pressures, 1 bar and 2 kbar. Error bars represent standard deviation.

**Fig. 2:**
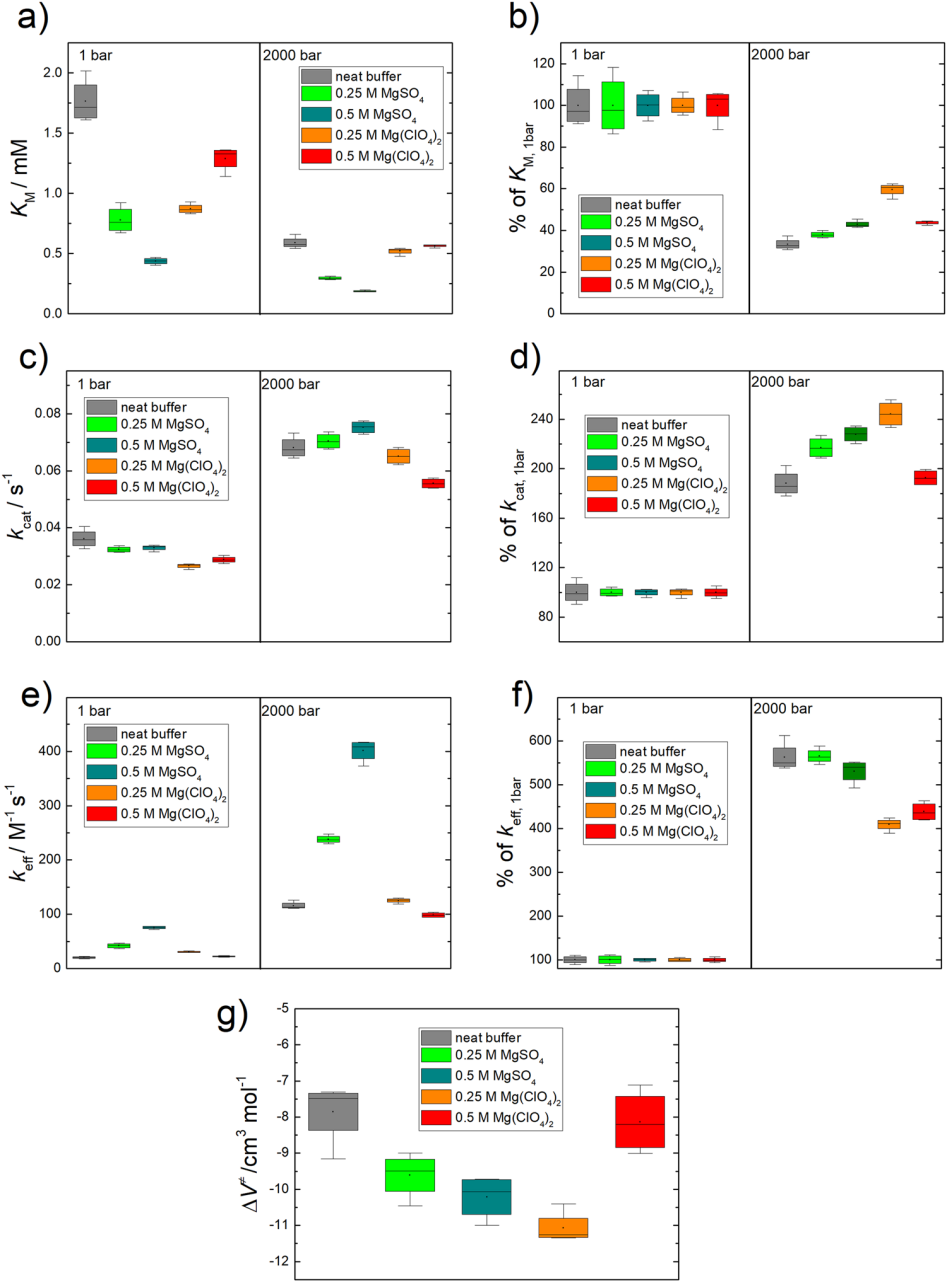
Kinetic and volumetric parameters of α-chymotrypsin activity. **a**, **c**, **e** Pressure dependence of the kinetic parameters (*K*_M_, *k*_cat_ and *k*_eff_ = *k*_cat_/*K*_M_) of the α-CT-catalysed hydrolysis reaction of SP*p*NA in different salt conditions. **b**, **d**, **f** Each salt condition was normalised to its own kinetic parameter at 1 bar (starting point: 100%). Activation volume at high substrate concentrations, reflecting (**g**) the volume change responsible for *k*_cat_. Error bars represent standard deviation.

The effects of pressure and high concentrations of Mars relevant salts on enzymes has not been previously investigated. The Michaelis–Menten plots in Fig. 1 show the activity of α-CT in the absence and presence of MgSO_4_ and Mg(ClO_4_)_2_. The activity of α-CT increases with pressure, as has been previously described^16–18^. Figure 2a, b show that the Michaelis constant, *K*_M_, decreases slightly with increasing pressure, i.e., the affinity of the substrate increases slightly upon compression. The turnover number, *k*_cat_, increases about 2–3 fold and the catalytic efficiency, *k*_cat_/*K*_M_ = *k*_eff_, rises by a factor of 5 in all solutions upon compression from 1 to 2000 bar. Table SI1 lists the kinetic parameters of *k*_cat_, *K*_M_ and *k*_cat_/*K*_M_ for α-CT in buffer in the absence and presence of MgSO_4_ and Mg(ClO_4_)_2_. The data show that the *k*_cat_ of α-CT is lower at each pressure step at both Mg(ClO_4_)_2_ concentrations than when in buffer alone. Despite the lower *k*_cat_ values, the reduced *K*_M_ values result in a greater catalytic efficiency (*k*_cat_/*K*_M_) for α-CT at 2000 bar in 0.25 M Mg(ClO_4_)_2_ compared to buffer, whereas the catalytic efficiency is reduced at 2000 bar in 0.5 M Mg(ClO_4_)_2_. The activity of α-CT is also increased in the presence of MgSO_4_ at both concentrations with greater *k*_cat_ values above 1 bar, lower *K*_M_ values, and greater catalytic efficiency.

The change in the kinetic parameters, in both real numbers and as percentages, are presented in Fig. 2a–f. The greatest proportional change in *k*_cat_ from 1 to 2000 bar is in the presence of 0.25 M Mg(ClO_4_)_2_ (+144%), the greatest proportional change in *K*_M_ is in buffer (−76.5%) and the greatest proportional change in *k*_cat_/*K*_M_ observed is in 0.25 M MgSO_4_ (+466%).

Figure 2g and Table SI2 show the data for the activation volume, which were determined using Eq. (2) (see below). All Δ*V*^≠^ values exhibit a negative value (order of magnitude, −8… −11 cm^3^ mol^−1^), which is similar to that described in the literature^19,20^. Furthermore, the Δ*V*^≠^ slightly increases with increasing substrate concentration (Table SI3), reaching plateau values for substrate concentrations of about 2 mM, i.e., for concentrations where the enzyme is saturated with substrate. The activation volume for large substrate concentrations, where *v*_0_ ∝ *k*_cat_, describes the difference in the volumes of the TS and the ES complex, i.e., Δ*V*^≠^ = *V*^≠^ − *V*_ES_, whereas measuring the activation volume at low substrate concentrations, where *v*_0_ ∝ *k*_cat_/*K*_M_, includes the volume change due to substrate binding, i.e., Δ*V*^≠^ = *V*^≠^ − *V*_E+S_^21,22^. The latter contribution seems to depend slightly on the particular solution conditions and amounts to about −4 to −6 cm^3^ mol^−1^, i.e., the ES substrate complex is slightly more compact relative to the partial volumes of enzyme and substrate, probably due to partial desolvation upon substrate binding. Δ*V*^≠^ values for all salt conditions reveal no significant differences compared to the neat buffer. Altogether, the TS is more compact than the ES complex, which might be due to a decrease of void volume and/or hydration of charges (electrostriction effect) in the TS.

The difference in the effect of both salts is largely attributable to the extent to which they preferentially interact with the peptide backbone (perchlorate) or are excluded from the peptide surface (sulphate)^23^. It is also worth noting that while this can be examined as an anion or cation effect, it is worth considering the whole salt due to the varied extent with which the ions pair with each other in water^24^.

### Pressure–temperature stability of α-chymotrypsin

**Fig. 3:**
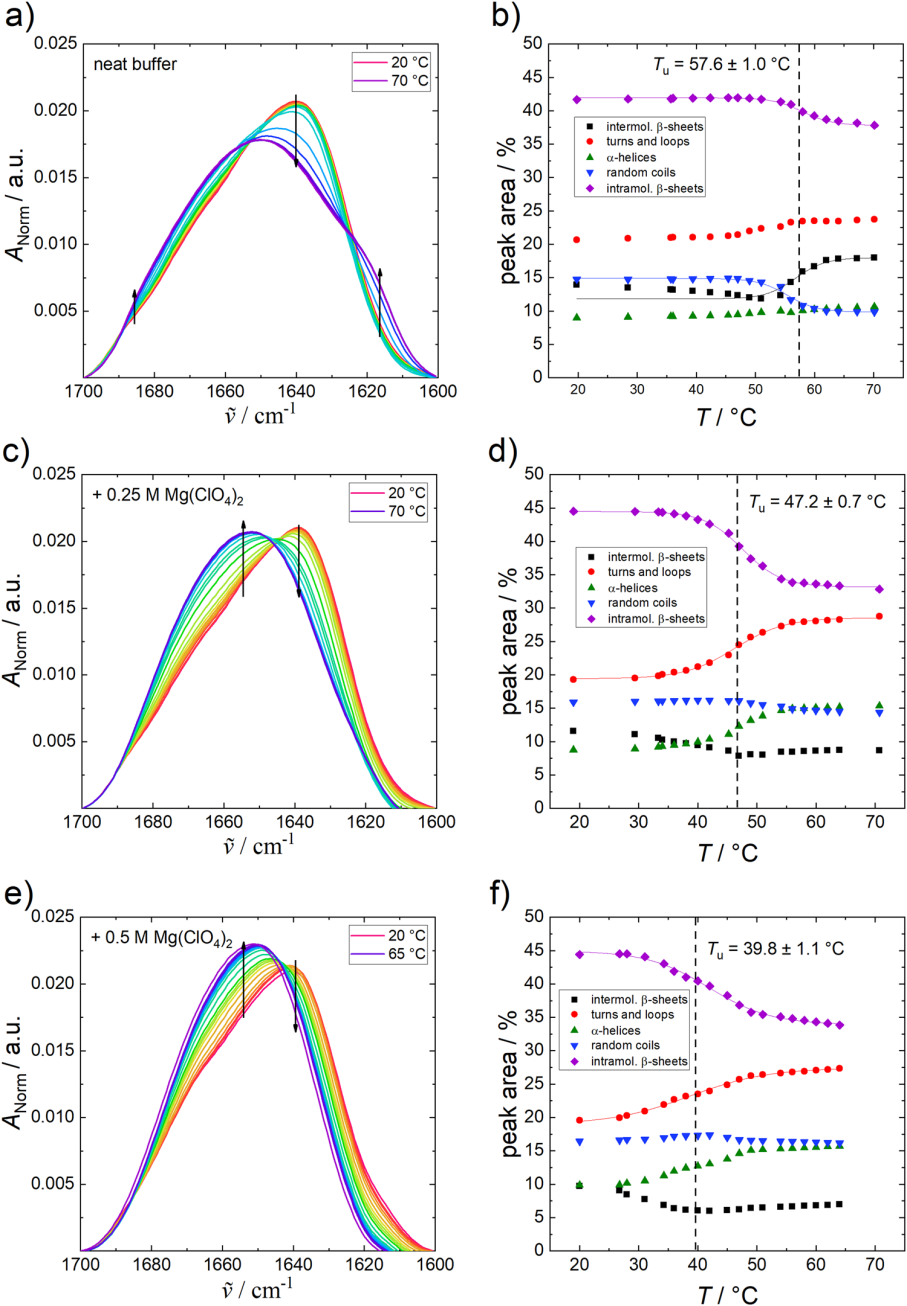
Changes to α-chymotrypsin FTIR spectra with temperature. Normalised temperature-dependent FTIR spectra of α-CT (50 mg mL^−1^) in neat buffer (+10 mM CaCl_2_) (**a**), 0.25 M (**c**) and 0.5 M Mg(ClO_4_)_2_ (**e**) at ambient pressure and the corresponding secondary structural changes (**b**, **d**, **f**) obtained from the curve fitting procedure. Arrows indicate increasing temperature and the lines represent the Boltzmann fits to the experimental data using Eq. (3).

To follow the temperature- and pressure-dependent unfolding process of α-CT in detail, the secondary structural changes in the presence of perchlorate compared to neat buffer (+10 mM CaCl_2_) were measured using Fourier-transform infrared (FTIR) spectroscopy, covering a temperature range from 20 to 70 °C and a pressure range from 1 bar to 10 kbar (1 GPa). Figure 3 displays the normalised temperature-dependent FTIR spectra of α-CT (Fig. 3a, c, e) as well as the corresponding changes in secondary structural elements (Fig. 3b, d, f) for different concentrations of perchlorate. At ambient pressure and 20 °C, the amide I´ band of α- CT in neat buffer shows a broad band at 1638 cm^−1^, with aggregation bands appearing at 1617 cm^−1^ and 1684 cm^−1^ at high temperatures. The secondary structure components obtained, which are derived from the curve fitting procedure (Fig. 3b), are in accord with earlier results reported by Meersman et al.^25^. At ambient pressure and 25 °C, α-CT shows high contents of intramolecular β-sheets (~43%) and turns and loops (~21%) and lower amounts of α-helices (~9%). These results are in rather good agreement with crystallographic data^26^. The minor differences between the X-ray diffraction and FTIR results (see Table SI 4) may be due to different absorption coefficients of the various secondary structure elements, which is not significant as only relative changes are essentially discussed here. Upon temperature-induced unfolding/denaturation, the intramolecular β-sheet content decreases (~−5%) and aggregation of the protein via formation of intermolecular β-sheets (~+5%) takes place in neat buffer (Fig. 3b).

Remarkably, in the presence of Mg(ClO_4_)_2_, no aggregation via formation of intermolecular β-sheets occurs upon unfolding at high temperatures. The intramolecular β-sheet content decreases and the α-helices & turns/loops content increases concomitantly. With increasing perchlorate concentration, the unfolding temperature, *T*_u_, of α-CT decreases from 57.6 °C to 39.8 °C, i.e., the salt leads to a marked destabilization of the temperature stability of the protein. Further, the width of the unfolding transition region increases upon addition of perchlorate, pointing to a decreasing cooperativity of the transition.

**Fig. 4:**
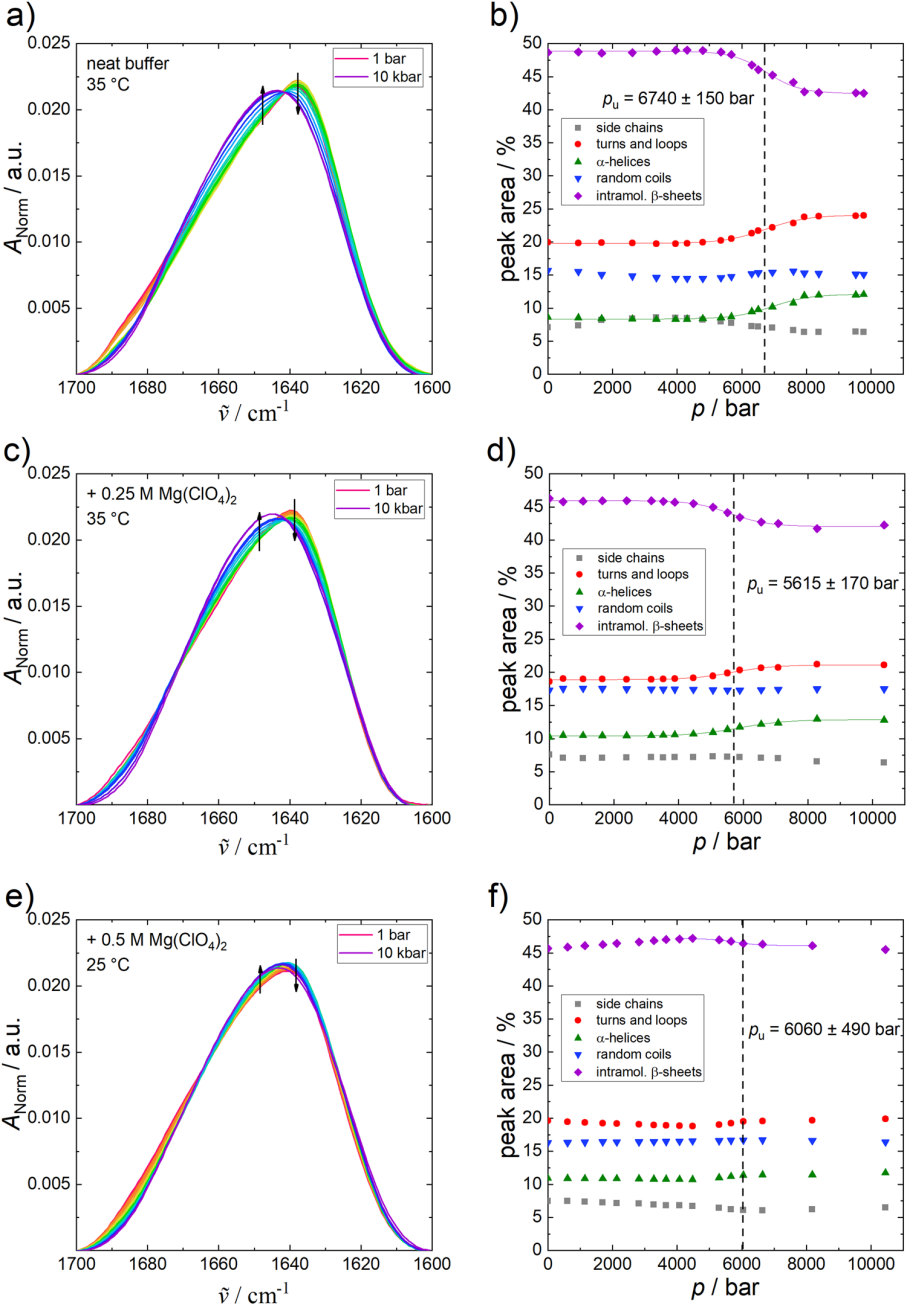
Changes to α-chymotrypsin FTIR spectra with pressure. Normalised pressure-dependent FTIR spectra of α-CT (50 mg mL^−1^) in (**a**) neat buffer (+10 mM CaCl_2_) (**a**), (**c**) 0.25 M Mg(ClO_4_)_2_ and (**e**) at 35 °C, 0.5 M Mg(ClO_4_)_2_ at 25 °C, and the corresponding secondary structural changes (**b**, **d**, **f**) obtained from the curve fitting procedure. Arrows indicate increasing pressure and lines show the Boltzmann fits to the experimental data using Eq. (4).

Figure 4 depicts the normalised pressure-dependent FTIR spectra of α-CT (Fig. 4a, c, e) and the corresponding secondary structural changes (Fig. 4b, d, f) at different concentrations of perchlorate at 35 °C up to 10 kbar. With increasing pressure in neat buffer solution, the amide I´ band of α-CT shifts to higher wavenumbers and partial unfolding is observed at around 6700 bar. Regarding the relative changes of secondary structure elements, the α-helix & turns/loops content increases (∼+4%), while the percentage of intramolecular β-sheets decreases (∼−4%) concomitantly upon compression. In the presence of 0.25 M Mg(ClO_4_)_2_, the secondary structural changes are similar, and α-CT partially unfolds at a lower pressure (5600 bar, 35 °C) compared to the buffer condition. In summary, the enzyme is very pressure stable, even in the presence of 0.25 M of perchlorate. The temperature- and pressure-induced (partial) denaturation is irreversible for all conditions measured.

Contrary to the conformational entropy gain of the peptide chain, which is the main driving force for the temperature-induced unfolding of proteins, volume changes and compressibility play a decisive role for the pressure-induced unfolding process, where the system tends to occupy an overall smaller volume state at high pressure^27,28^. To gain a quantitative measure, the volume change of (partial) unfolding, Δ*V*_u_, was determined using Eq. (4). For the partial unfolding of α-CT at 35 °C in the absence of Mg(ClO_4_)_2_, a value of Δ*V*_u_ = −41 ± 7 cm^3^ mol^−1^ was obtained, which is consistent with the volume change of the precursor (chymotrypsinogen) of α-CT^29^ and a typical value observed for unfolding of monomeric proteins^28,30–32^. At lower temperatures (25 °C) or higher perchlorate concentrations (0.5 M), smaller changes in the secondary structure elements are observed upon pressure-induced unfolding, which is reflected in smaller Δ*V*_u_ values.

**Fig. 5:**
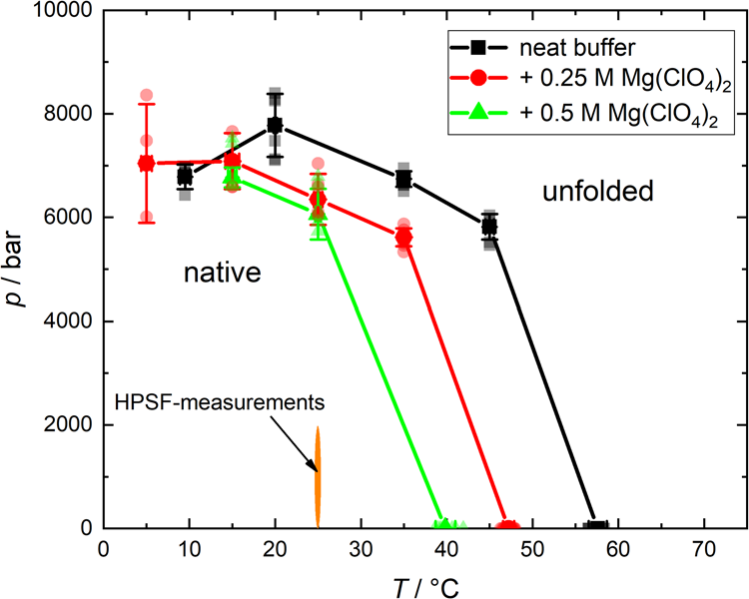
*p, T*-stability phase diagram of α-chymotrypsin. *p*, *T*-stability phase diagram of α-chymotrypsin (50 mg mL^−1^, 10 mM CaCl_2_). The lines indicate the transition curve, where Δ*G*_u_ = 0, i.e., show the transition from the native to the (partially) unfolded state. The kinetic high-pressure stopped-flow (HPSF) measurements were performed in a region where the protein is well within the natively folded state. Error bars represent standard deviation.

The *p*, *T*-phase diagram of α-CT in the absence and presence of Mg(ClO_4_)_2_, resulting from these temperature- and pressure-dependent FTIR measurements, is depicted in Fig. 5. At low temperatures and pressures, the native state of the protein is favoured. Crossing the transition line to the unfolded state, the native conformation loses its stability and the protein partially unfolds, losing its enzymatic activity. As is clearly seen from the figure, increasing the perchlorate concentration reduces the pressure and temperature stability of the protein, shifting the boundary to lower values of temperature and pressure. However, the enzyme is stable in the whole *p, T-*range covered in the HPSF studies, and also in the presence of the two salts.

Literature data on the phase diagram of the α-CT only exists for solution conditions different from ours. The protein unfolds in the absence of Ca^2+^ at 4.9 kbar for *T* = 21 °C and at 41 °C for ambient pressure conditions^33^. As expected, Ca^2+^ ions have a positive effect on the temperature and pressure stability of the protein, probably by electrostatic screening of negatively charged surface patterns of the protein. A stabilising effect of Ca^2+^ ions on α-CT has been observed in other unfolding studies of proteins as well^34,35^. A marked destabilising effect of perchlorate, being a typical low-charge-density anion at the far end of the Hofmeister series of anions, at high concentrations, has been observed in lysozyme at pH 7 as well and is expected to be due to weak binding of the ClO_4_^−^ anions to the protein^36,37^. This reduces the Gibbs energy associated with hydrating of the newly exposed interior of the protein upon unfolding, thereby destabilising the native protein’s fold. As has been shown recently by Dougan et al.^38^, the water structure also changes markedly at the high perchlorate concentrations mimicking conditions in Martian soil. They found that the tetrahedral structure of water is heavily perturbed, the effect being equivalent to pressurising pure water to pressures of the order of 20 kbar and more. Interestingly, the Mg^2+^ and ClO_4_^−^ ions appear charge-ordered and bridged by water molecules under these conditions, thereby preventing ice formation at low temperatures.

## Discussion

Little is known about the effects of high ion concentrations on cellular life or the combined effects of ions with high pressure. Yet these combinations can shape the habitability of deep subsurface environments on Earth and potentially elsewhere. Currently, one scientific question of great interest is the habitability of the Martian subsurface. Although the question of whether there is life on Mars remains speculative, we can ask questions about how physical and chemical conditions expected on Mars might theoretically influence its habitability. We can then use terrestrial organisms and biomolecules to explore the boundary space of habitability for known life under said Martian conditions. In this study, we used a well characterised enzyme to investigate the combined effects of perchlorate ions and high pressures as a general proxy for biomolecular stability under these extremes.

From the Michaelis–Menten plots it is demonstrable that increasing the hydrostatic pressure also increases the enzyme activity of α-CT, even in the presence of high concentrations of the chaotropic salt Mg(ClO_4_)_2_. As α-CT has a negative activation volume (Δ*V*^≠^ < 0), the increasing activity with pressure is largely attributed to Le Châtelier’s principle as previously described^16–18^.

From the kinetic parameters it is also shown that Mg(ClO_4_)_2_ reduces the activity of α-CT at both concentrations, 0.25 and 0.5 M, and across all pressure conditions tested. This agrees with previous ambient pressure studies showing the same effect of perchlorate salts on α-CT and other enzymes^7,13^. Furthermore, the observation that MgSO_4_ increases the activity of α-CT agrees with previous studies which show that the sulphate ion can stabilise proteins^14^ and increase the activity of α-CT^15^. This sulphate-induced effect is also shown to be substrate dependent as it was absent in a previous kinetic study using *N*-benzoyl-L-tyrosine ethyl ester as the substrate.

Different proportional increases in *k*_cat_ were observed in response to increasing hydrostatic pressure for the different salt conditions. From previous work at atmospheric pressure it has been shown that perchlorate salts reduce the structural stability of α-CT. It may therefore be the case that high pressures can partially undo this negative structural effect, manifesting itself as the greater proportional increase in *k*_cat_. In essence, lesser salt-induced structural disruption at higher pressures may confer greater than expected activity increases than due to Le Châtelier’s principle alone. This suggests the existence of an advantageous salt-pressure-activity interplay.

The shift of the α-CT pressure-temperature stability diagram to lower pressures and temperatures with increasing Mg(ClO_4_)_2_ concentration also suggests that the region of peak stability is concomitantly shifting to the left also. Therefore, in the presence of Mg(ClO_4_)_2_, the region of peak α-CT activity and stability may be found at elevated pressures, and lower temperatures.

It is of interest to note that the pressure-temperature phase diagram of life resembles that of proteins, which is due to the particular temperature dependence of the proteins’ specific heat of unfolding^39^_._ Life and proteins both exhibit similar, curved pressure-temperature phase diagrams, whereas lipids and nucleic acids generally have linear pressure-temperature phase diagrams^28,40,41^. If we were to imagine a *z*-axis emerging from Fig. 5 representing the (standard) free energy of unfolding (Δ*G*_u_), we would be able to see the protein stability phase space as a function of free energy also. In this phase diagram, α-CT would exhibit a peak of stability (maximum of Δ*G*_u_) which descends down to the measured unfolding pressures and temperatures where Δ*G*_u_ = 0. It would be interesting to know how the perchlorate salts affect the position of the region of peak stability. If it was found that the perchlorate salt moves the region of peak stability to higher than ambient pressures and lower temperatures, is it also moving the region of peak fitness for life? If so, it would suggest that life is more favourable in perchlorate brines if they also experience increased pressures and lower temperatures (a similar salinity-pressure–temperature-growth relationship has been described by Kaye and Baross^42^). It is then of interest that such environments have either been potentially observed or theorised to be present on Mars^2,3^. Therefore, whilst concentrated perchlorate brines reduce the habitability of an aqueous environment, high pressures and lower temperatures may counteract the deleterious perchlorate effect, thus increasing habitability. There is already tentative evidence showing that sub 0 °C temperatures confer greater survival of bacteria in perchlorate brines^43^.

While the effects of low temperatures on our model enzyme in the presence of perchlorate salts remains unknown, we have shown that high pressures can still increase enzyme activity even in the presence of a destabilising salt predicted to be concentrated on Mars. Moreover, high pressures may confer greater structural stability, reducing the destabilising effects of perchlorate salts on proteins. It would be of particular interest to expand our understanding of the perchlorate-pressure-temperature effect on membrane proteins as, in addition to secreted biomolecules, they would be directly exposed to the perchlorate-rich environment. Thus, the effect that concentrated perchlorate salts exert on processes such as molecular transport and signalling may be of particular importance to habitability. Finally, similar to the case of the enzymes of extremely halophilic archaea^44^, changes in amino acid composition under such high Mg(ClO_4_)_2_ concentrations, e.g., by changing the ratio of negatively to positively charged and hydrophobic residues^44^, might help in optimising enzymatic activity under such harsh salt concentrations.

## Methods

### Materials

Lyophilised α-chymotrypsin from bovine pancreas, Tris (Tris(hydroxymethyl)aminomethane) as well as the salts MgSO_4_, Mg(ClO_4_)_2_ and CaCl_2_ were obtained from Sigma-Aldrich, Germany.

### Measuring α-chymotrypsin activity

Enzyme activity was measured using a high-pressure stopped-flow system, HPSF-56, from Hi-Tech Scientific^16,19,45^. The enzyme was prepared as a 20 µM buffered stock solution (0.1 M Tris- HCl, 0.01 M CaCl_2_, pH 7.8) in the absence or presence of MgSO_4_ or Mg(ClO_4_)_2_ at either 0.25 or 0.5 M. Enzyme concentration was measured using its absorbance at 280 nm with a molar extinction coefficient of 51000 M^−1^ cm^−1^. Stock solutions of *N*-Succinyl-Phe-*p*-nitroanilide (SP*p*NA) were prepared at 8 mM in the same buffer and salt concentrations as enzyme stock solutions. The enzyme concentration was maintained at 10 µM post-mixing for all activity assays with post-mixing substrate concentrations of 0.25, 0.5, 1, 2 and 4 mM. The pressures assayed were 1, 500, 1000, 1500 and 2000 bar. The reaction was monitored for 50 s post-mixing as the change in absorbance at 410 nm due to the formation of the reaction product *p*-nitroaniline, which has a molar extinction coefficient of 8800 M^−1^ cm^−1^. Temperature was controlled and maintained at 20 °C by a thermostat.

The enzyme activity was plotted in Origin 9.3 (OriginLab) and a least-squares fit fitting method was applied according to the Michaelis–Menten equation:

1 $$v \,=\, \frac{{v_{{\mathrm{max}}}[{\mathrm{S}}]}}{{K_{\mathrm{M}} + [{\mathrm{S}}]}}$$

The Eyring-equation describes the pressure effect on the rate of the reaction^46^:

2 $$\left( {\frac{{\partial \ln (k{\mathrm{/}}k_0)}}{{\partial p}}} \right)_T \,=\, - \frac{{{\Delta} V^ \ne }}{\mathrm\it{{{RT}}}}$$

(*p* = pressure, *T* *=* temperature, *R* = ideal gas constant, Δ*V*^≠^ = activation volume, *k*_0_ = rate constant at a reference pressure, 1 bar). Within the framework of the Michaelis–Menten formalism, *k* = *k*_cat_, when the substrate concentration is large, and *k* = *k*_cat_/*K*_M_, when the substrate concentration, [S], is small. The activation volume describes the difference of the volume of the TS and the ground state (ES) of the ES complex or the reactants E and S. Due to different compressibilities of the reactants, the ES complex and the TS, Δ*V*^≠^ values can be positive or negative, and they may also be dependent on the pressure amplitude.

### FTIR: Sample preparation and secondary structure analysis

The enzyme α-chymotrypsin from bovine pancreas was purchased from Sigma-Aldrich as lyophilized powder. For H/D-exchange, the protein was dialysed against D_2_O using Amicon Ultra (2 mL) centrifugation units with 10 kDa cut-off and subsequently lyophilized. For the pressure dependent FTIR spectroscopy studies, the measurements were carried out in 100 mM Tris (Tris(hydroxymethyl)aminomethane) buffer with 10 mM CaCl_2_ in the absence and in the presence of Mg(ClO_4_)_2_. For the temperature-dependent experiments, phosphate buffer was used. The pD-value of both buffers was adjusted to 7.8 (pH + 0.4 = pD) by adding DCl. All chemicals were used without further purification.

FTIR spectra were collected using a Nicolet 6700 (Thermo Fisher Scientific) equipped with a liquid-nitrogen cooled MCT-detector (HgCdTe), operated at –196 °C, in the wavenumber range between 4000 and 650 cm^−1^. The sample chamber was continuously purged with CO_2_-free and dry air. All measured spectra were averaged over 128 scans in a row at a spectral resolution of 2 cm^−1^ and were processed with Happ-Genzel apodization by using Omnic 7.2 spectral processing software. The equilibration time before each spectrum was recorded at each temperature was 15 min, for the pressure dependent studies, 5 min. The temperature IR cell consists of two CaF_2_ windows separated by a mylar spacer of 50 µm thickness. The setup of the pressure system consists of a membrane-driven diamond anvil cell (VivoDac Diacell^®^) with type IIa diamonds, which is connected to an automated pneumatic pressure control (Diacell^®^ iGM Controller, Almax easyLab). To measure the pressure inside the cell, by adding BaSO_4_, the pressure-sensitive stretching vibration of SO_4_^2−^ (∼983.5 cm^−1^ ≙ 1 bar, 25 °C) was used as an internal pressure calibrator^47^. For each measurement, a protein concentration of 5 wt.% was used and the temperature of the cell was regulated with an external, circulating water thermostat. Processing and analysis of the spectra was carried out with the Grams AI 8.0 software (Thermo Fisher Scientific). Depending on the sample, spectra of the buffer systems were subtracted from the spectra recorded and smoothed afterwards. Then, the area of the amide I´ band (1700–1600 cm^−1^, which is essentially based on the conformation sensitive C=O stretching vibration of the peptide bonds) was normalised to 1. Due to small variations in molecular geometry and hydrogen bonding patterns, it is possible to analyse the secondary structural composition and conformational changes of proteins^48^. The number of subbands and their positions for fitting were obtained via Fourier self-deconvolution (FSD) and 2nd derivative approaches. Eight subbands, similar to the results described in the literature^25^ were obtained and mixed Gaussian–Lorentz functions were used to fit the peak areas in the amide I´ band region and to determine the relative changes in the population of secondary structure elements (curve fitting)^49^. Assuming a two-state unfolding process of the protein, a Boltzmann function can be fitted to the temperature- and pressure-dependent sigmoidal curve progression of the intensity changes:

3 $$I \,=\, \frac{{I_{\mathrm{f}} - I_{\mathrm{u}}}}{{1 + {\mathrm{e}}^{ - \left( {\frac{1}{{T_{\mathrm{u}}}} - \frac{1}{T}} \right) \cdot \frac{{{\Delta} H_{{\mathrm{vH}},{\mathrm{u}}}}}{R}}}} + I_{\mathrm{u}},$$

4 $$I \,=\, \frac{{I_{\mathrm{f}} - I_{\mathrm{u}}}}{{1 + {\mathrm{e}}^{ - \left( {p - p_{\mathrm{u}}} \right) \cdot \frac{{{\Delta} V_{\mathrm{u}}}}{{RT}}}}} + I_{\mathrm{u}},$$

where *I*_f_ and *I*_u_ are the plateau values of the IR band intensities, *I*, of the folded and unfolded protein. The unfolding temperature, *T*_u_, and unfolding pressure, *p*_u_, were obtained from the inflection points of the sigmoidal curves. In addition, the thermodynamic parameters ${\Delta} H_{{\mathrm{vH}},{\mathrm{u}}}$ and Δ*V*_u_, i.e., the corresponding van´t Hoff enthalpy and volume changes, respectively, can be directly obtained from the fits of the temperature- and pressure-dependent FTIR data.

### Statistics and reproducibility

The enzyme activity results represent two biological replicates, each made of two technical replicates. The structural FTIR results represent triplicate measurements. No data points were excluded from the respective analyses.

### Reporting summary

Further information on research design is available in the Nature Research Reporting Summary linked to this article.

## Supplementary information

Supplementary Information

Description of Additional Supplementary Files

Supplementary Data 1

Reporting Summary

## Data Availability

The source data for the figures presented in this study have been included as a supplementary file.
