# Supplementary material for: High pressures increase α-chymotrypsin enzyme activity under perchlorate stress
Source: Commun Biol. 2020 Oct 2;3:550. doi: 10.1038/s42003-020-01279-4 (PMC7532203; doi:10.1038/s42003-020-01279-4)
Supplement: Supplementary file 1 — Supplementary Information [file 42003_2020_1279_MOESM1_ESM.pdf]

Supporting Information

Table SI1: Calculated kinetic parameters (Michaelis constant (K_M), turnover number (k_{cat}) and catalytic efficiency ($k_{cat}/K_M = k_{eff}$)) of the α-CT reaction for all pressures and solution conditions (T = 20 °C). Errors presented are the SD.

Buffer solution	p / bar	K_M / mM	k_{cat} / s^{-1}	k_{cat}/K_M / s^{-1} M^{-1}
Tris buffer	1	1.756 ± 0.207	0.0361 ± 0.0020	20.567 ± 2.663
	500	1.038 ± 0.199	0.0409 ± 0.0030	39.372 ± 8.084
	1000	0.855 ± 0.134	0.0477 ± 0.0027	55.715 ± 9.290
	1500	0.670 ± 0.070	0.0559 ± 0.0019	83.427 ± 9.170
	2000	0.588 ± 0.068	0.0680 ± 0.0025	115.573 ± 14.102
+ 0.25 M $MgSO_4$	1	0.769 ± 0.088	0.0323 ± 0.0013	42.018 ± 5.102
	500	0.600 ± 0.074	0.0420 ± 0.0016	69.974 ± 9.026
	1000	0.417 ± 0.058	0.0474 ± 0.0018	113.770 ± 16.340
	1500	0.295 ± 0.018	0.0539 ± 0.0018	182.610 ± 12.857
	2000	0.296 ± 0.036	0.0704 ± 0.0021	237.876 ± 30.133
+ 0.5 M $MgSO_4$	1	0.438 ± 0.159	0.0330 ± 0.0016	75.392 ± 13.062
	500	0.330 ± 0.070	0.0437 ± 0.0023	132.299 ± 28.828
	1000	0.258 ± 0.049	0.0498 ± 0.0021	193.376 ± 37.344
	1500	0.221 ± 0.035	0.0612 ± 0.0021	277.317 ± 45.021
	2000	0.187 ± 0.028	0.0752 ± 0.0022	401.408 ± 60.324
+ 0.25 M $Mg(ClO_4)_2$	1	0.872 ± 0.159	0.0267 ± 0.0018	30.551 ± 5.912
	500	0.826 ± 0.143	0.0383 ± 0.0024	46.332 ± 8.520
	1000	0.675 ± 0.073	0.0434 ± 0.0016	64.266 ± 7.363
	1500	0.593 ± 0.050	0.0530 ± 0.0014	89.393 ± 7.878
	2000	0.520 ± 0.048	0.0651 ± 0.0018	125.073 ± 11.951
+ 0.5 M $Mg(ClO_4)_2$	1	1.287 ± 0.161	0.0288 ± 0.0015	22.396 ± 3.030
	500	0.899 ± 0.099	0.0358 ± 0.0014	39.811 ± 4.666
	1000	0.766 ± 0.078	0.0408 ± 0.0013	53.340 ± 5.715
	1500	0.621 ± 0.052	0.0470 ± 0.0013	75.641 ± 6.639
	2000	0.564 ± 0.032	0.0556 ± 0.0010	98.450 ± 5.899

Table SI2: Calculated activation volumes, $\Delta V^{\neq}$, for the hydrolysis of SP*p*NA catalysed by α-CT at different solution conditions (T = 20 °C). Errors presented are the SD.

Buffer solution	$\Delta V^{\neq}$ / cm^3 mol^{-1}
Tris buffer	-7.8 ± 0.4
+ 0.25 M $MgSO_4$	-9.0 ± 0.8
+ 0.5 M $MgSO_4$	-9.9 ± 0.6
+ 0.25 M $Mg(ClO_4)_2$	-10.5 ± 1.0
+ 0.5 M $Mg(ClO_4)_2$	7.9 ± 0.4

Table SI3: Calculated activation volumes, $\Delta V^{\neq}$, of the α-CT reaction for each substrate concentration at different solution conditions (T = 20 °C). Errors presented are the SD.

	$c_{Substrate}$ /mM	$\Delta V^{\neq}$ / cm^3 mol^{-1}
Tris buffer	0.25	-16.8 ± 2.5
	0.5	-16.2 ± 0.7
	1	-14.0 ± 0.9
	2	-12.3 ± 0.7
	4	-10.5 ± 0.3
+ 0.25 M $MgSO_4$	0.25	-16.6 ± 0.6
	0.5	-14.8 ± 0.9
	1	-13.3 ± 0.6
	2	-11.6 ± 0.6
	4	-10.4 ± 0.9
+ 0.5 M $MgSO_4$	0.25	-14.6 ± 1.2
	0.5	-13.2 ± 0.6
	1	-12.7 ± 0.8
	2	-11.3 ± 0.5
	4	-10.5 ± 0.7
+ 0.25 M $Mg(ClO_4)_2$	0.25	-15.6 ± 1.8
	0.5	-15.3 ± 1.0
	1	-11.8 ± 0.3
	1.5	-12.7 ± 0.7
	3	-11.5 ± 1.1
+ 0.5 M $Mg(ClO_4)_2$	0.25	-14.1 ± 1.6
	0.5	-13.7 ± 0.8
	1	-13.1 ± 1.4
	2	-10.5 ± 0.7
	4	-9.7 ± 0.7

Table SI 4: Quantitative estimation of the secondary structure content of α-CT by FTIR analysis compared with literature data by different methods.

Protein	Method	α-helices (%)	β-sheets (%)	random coils (%)	turns and loops (%)
α-Chymotrypsin	FTIR	9	42	15	22
	X-Ray (1)	9	34	57*	
	X-Ray (2)	8	50	15	27
	FTIR (3)	14	47	14	30
	FTIR (4)	11	49	18	21

*Remaining percentage.

1) Byler, M. and Susi H., Examination of the Secondary Structure of Proteins by Deconvolved FTIR Spectra, Biopolymers, **25**, 469–487 (1986).

2) Levitt, M. & Greer, J. Automatic identification of secondary structure in globular proteins. *J. Mol. Biol.* **114**, 181–293 (1977).

3) Qinglong, C. Huizhou, L. and Jiayong, C. Fourier transform infrared spectra studies of protein in reverse micelles: effect of AOT/isooctane on the secondary structure of a-chymotrypsin, *Biochim. Biophys. Acta*, **1206**, 247–252 (1994).

4) Meersman, F., Dirix, C., Shipovskov, S., Klyachko, N. L. & Heremans, K. Pressure-induced protein unfolding in the ternary system AOT-octane-water is different from that in bulk water. *Langmuir* **21**, 3599–3604 (2005).
