# Supplementary material for: High pressures increase α-chymotrypsin enzyme activity under perchlorate stress
Source: Commun Biol. 2020 Oct 2;3:550. doi: 10.1038/s42003-020-01279-4 (PMC7532203; doi:10.1038/s42003-020-01279-4)
Supplement: Supplementary file 2 — Description of Additional Supplementary Files [file 42003_2020_1279_MOESM2_ESM.pdf]

Description of Additional Supplementary Files

File Name: Supplementary Data 1
Description: Source data for all the manuscript's graphs.
